# Maternal Emotion Coaching and Child Emotion Regulation: Within-Interaction Sequences in Early Childhood

**DOI:** 10.1007/s42761-024-00285-7

**Published:** 2025-01-11

**Authors:** Danhua Zhu, Fantasy T Lozada, Cynthia L Smith, Martha Ann Bell, Julie C Dunsmore

**Affiliations:** 1 University of California, CA Irvine, 9269 USA; 2 Virginia Commonwealth University, 907 Floyd Ave, Richmond, VA 23284 USA; 3 Virginia Tech, Blacksburg, VA 24061 USA; 4 University of Houston, Houston, 4302 University Dr, TX 77004 USA

**Keywords:** Emotion coaching, Emotion regulation, Early childhood, Within-interaction sequences, Observational

## Abstract

Emotion socialization is a dynamic transactional process that unfolds at the moment during parent-child interactions. To better understand these transactions (both parent-driven and child-driven) in early childhood, we conducted a lag-sequential analysis examining sequential contingency between maternal emotion coaching and child emotion regulation at ages 3 and 4 years. Mother-child dyads in the southeastern United States (*N* = 208 for age 3 timepoint [101 boys, 107 girls] and 227 for age 4 timepoint [115 boys, 112 girls]) participated in a laboratory etch-a-sketch task, which was videorecorded and later observationally coded for maternal coaching of both positive and negative emotions and for child emotion regulation (indexed as compliance, engagement, and low frustration) at 30-s intervals. At age 3, we found two reciprocal sequences: (1) When mothers coached positive emotions, children were subsequently more likely to show compliance, and when children complied, mothers were subsequently more likely to coach their positive emotions; (2) when mothers coached negative emotions, children were subsequently more likely to display frustration, and when children showed frustration, mothers were subsequently more likely to coach their negative emotions. At age 4, we only found parent-driven, positive emotion–related sequences: when mothers coached positive emotions, children were subsequently more likely to show compliance and engagement. Findings shed light on the distinct functions of positive and negative emotions as well as the intricacy of dynamic emotion socialization transactions in relation to child emotion regulation during early childhood.

**Supplementary Information:**

The online version contains supplementary material available at 10.1007/s42761-024-00285-7.

Emotion socialization refers to the process through which socializers influence children’s experience, communication, and regulation of emotions (Eisenberg, 2020). Our study focused on parental emotion coaching, which shows acceptance of children’s emotion, encourages emotional expression, and assists with problem-solving the emotion-eliciting situation (Gottman et al., 1996). When parents engage in coaching of children’s negative emotions, children show better emotion regulation, which in turn relates to children’s better adaptive functioning (Katz et al., 2012). Parents’ coaching of children’s positive emotions has been less studied. However, findings with adolescents’ physiological regulation and preschool-age children’s internalizing behavior suggest benefits of parental coaching of positive emotions for children’s emotion regulation (Hernandez et al., 2018; Miller-Slough & Dunsmore, 2023). Despite this body of research, a gap remains regarding how emotion coaching relates to children’s emotion regulation within interactions. Theories describe emotion socialization as a dynamic process that unfolds during parent–child interactions, and innovative microanalytic studies have examined how temporal relations of parent and child emotional expressions and behavioral responses relate to child outcomes (Eisenberg, 2020; Lougheed et al., 2016; Lunkenheimer et al., 2020; Wang, 2023). Yet, contingent relations between parental emotion coaching and child emotion regulation within interaction had not been previously investigated, perhaps because the process of emotion coaching is not captured by point or momentary behaviors and requires consideration of pacing within the flow of the interaction (Kehoe et al., 2020). We bridged this gap by observing maternal emotion coaching and child emotion regulation during 30-second intervals throughout a laboratory task and sequentially analyzing their relations.

Extant literature largely focuses on parental socialization of negative emotions as negative emotions may be challenging for younger children to cope with on their own (Hooven et al., 1995) and may impact children’s psychological and behavioral outcomes (Daniel et al., 2020). Meanwhile, emerging studies suggest an important social function of positive emotions in reducing behavioral problems and fostering children’s self-regulation (e.g., Shin et al., 2023; Yi et al., 2016). In a recent narrative review, Doan et al. (2023) emphasized the promising effect of positive emotions on child physical and mental health as well as the scarcity of research on the socialization of positive emotions. Accordingly, we assessed maternal coaching of both positive and negative emotions.

Based on theory and empirical findings, we studied maternal emotion coaching in relation to child emotion regulation behaviors to capture their within-interaction sequences. Eisenberg’s (2020) emotion socialization model proposed that parental emotion socialization behaviors influence children’s emotional arousal and regulation subsequently as well as over time. Gottman et al. (1996) viewed families’ emotional life and child development via parental meta-emotion philosophy—parents’ organized thoughts, beliefs, and approaches to their own and their children’s emotions—and theorized that parental emotion coaching meta-emotion philosophy impacts child emotion regulation via regulatory physiology. Research showed concurrent and longitudinal associations of maternal emotion coaching with preschoolers’ emotion regulation (Ellis et al., 2014; Wu et al., 2022). Eisenberg’s (2020) model also proposed that children’s emotion regulation influences their socially competent behaviors, which then subsequently alters the situational context that informs parental emotion socialization behaviors. Research demonstrated that when children were high in self-regulation and effortful control (a temperamental construct that relates to emotion regulation), mothers decreased over time in negativity (Klein et al., 2018) and increased over time in supportive responses (Feng et al., 2017). One thing to notice is that all the reviewed studies took a macroscopic approach in creating summary scores across the session and testing their relations to other variables of interest. When we are interested in process—how behaviors unfold in sequence, which is the case for maternal emotion coaching and child emotion regulation transactions—using a microscopic approach such as sequential analysis is more appropriate (Bakeman & Quera, 2011). Sequential analysis allows us to address process questions concerning contingency and can be applied to a sample of any size (Yoder & Tapp, 2004).

Maternal emotion coaching and child emotion regulation both include nonverbal as well as verbal components, making behavioral observations advantageous (Bakeman & Quera, 2011). Researchers often observe emotion socialization via parent–child reminiscing conversations about emotional events (e.g., Hernandez et al., 2018; Wu et al., 2022). Although emotion discourse tasks provide parents opportunities to intentionally reference, discuss, and respond to emotions, such intentions may be relatively uncommon during families’ daily lives—especially during the early childhood years that are packed with regular routines (Wildenger et al., 2008). Thus, we observed maternal emotion coaching and child emotion regulation during an etch-a-sketch task in which mothers and children collaboratively drew various shapes including a square (lower difficulty level) and a house/circle (higher difficulty level). As we did not directly ask dyads to engage with emotions, the etch-a-sketch task with varied difficulty levels enabled us to observe mothers’ unprompted, spontaneous coaching responses to their child’s positive (e.g., pride, excitement) and negative (e.g., frustration, anger) emotions that arose during the task (Zhu et al., 2024). Emotion regulation may be defined as the ability to display socially and contextually appropriate behaviors when experiencing stressful or emotional situations (Morris et al., 2007). Given the context of the etch-a-sketch task, the behaviors indexing emotion regulation included children’s behavioral compliance, engagement, and low expressed frustration.

Our objective was to examine parent-driven and child-driven contingencies for a more holistic understanding of the socialization process during dyadic interactions. Parent-driven contingencies (Aim 1) pertained to how mothers’ emotion coaching related to their child’s subsequent emotion regulation, whereas child-driven contingencies (Aim 2) concerned the reverse, how child emotion regulation related to subsequent maternal emotion coaching. According to the aforementioned literature, we expected children to show more emotion regulation behavior (more compliance, more engagement, and less frustration) following mothers’ emotion coaching. We also expected that following children’s emotion regulation behavior (compliance, engagement, or low frustration), mothers would show more emotion coaching behavior. Positive and negative emotions serve different functions in socialization and self-regulatory processes (Ramakrishnan et al., 2019; Tice et al., 2004). However, we did not specify hypotheses regarding positive versus negative emotions for both aims due to the scarcity of relevant empirical studies.

## Method

### Participants

Participants of the current study represent a subset of 410 mother–child dyads recruited when children were infants in a small college town and a mid-size city in the southeastern United States for a longitudinal study of emotion and cognition across early development (i.e., the CAP study; e.g., Bruce et al., 2023). The current sample includes dyads who completed a videorecorded interaction task during their lab visits at age 3 years and/or age 4 years. At age 3 years, 217 mother–child dyads initially completed the interaction task. Nine dyads were excluded due to video or timestamp issues that precluded matching 30-second intervals between types of codes. Compared to dyads who were included, dyads who were excluded had mothers with higher educational level (*t*(404) = 2.52, *p* = .012) with no other demographic differences (*p*s ≥ .127). The final sample size for the 3-year-old timepoint was 208 mother–child dyads (101 boys, 107 girls). Children were identified by mothers as White (75.5%), Black or African American (17.3%), or multi-racial or from other racial groups (7.2%). Most children were identified by mothers as not Hispanic (92.8%). Maternal education was as follows: 2.4% did not complete high school, 26.9% obtained a high school degree, 7.2% completed technical school, 43.3% completed college, 18.3% obtained a graduate school degree, and 1.9% of mothers’ highest level of education was missing.

At age 4 years, 261 mother–child dyads initially completed the same interaction task. Thirty-four dyads were excluded due to video or timestamp issues that precluded matching 30-second intervals between types of codes (see “Data Screening and Reduction”). There were no demographic differences between dyads who were included and those who were excluded (*p*s ≥ .067). The final sample size for the 4-year-old timepoint was 227 mother–child dyads (115 boys, 112 girls). Sample ethnic-racial and maternal education representation was similar to the 3-year-old timepoint. Table 1 shows the full demographics for each timepoint. One hundred eighty-one mother–child dyads were included at both timepoints, which was 87.0% of the 208 dyads at age 3 years and 79.7% of the 227 dyads at age 4 years.

**Table 1:** Participating dyads’ mother-reported demographic information at 3-year-old and 4-year-old timepoints

		**3 years (*n* = 208)**		**4 years (*n* = 227)**	
		*N*	Percentage	*N*	Percentage
Child gender	Boys	101	48.6%	115	50.7%
	Girls	107	51.4%	112	49.3%
Child race	White	157	75.5%	173	76.2%
	Black/African American	36	17.3%	36	15.9%
	Multi-racial or other	15	7.2%	18	7.9%
Child ethnicity	Hispanic	15	7.2%	18	7.9%
	Not Hispanic	193	92.8%	209	92.1%
Maternal educational level^a^	Some high school	5	2.4%	4	1.8%
	High school	56	26.9%	58	25.6%
	Technical school	15	7.2%	14	6.2%
	College	90	43.3%	97	42.7%
	Graduate school	38	18.3%	50	22.0%
	Missing	4	1.9%	4	1.8%

^a^At child’s birth

### Procedure

The institutional review board at the universities in each research location approved all procedures for the original study. Informed consent was obtained from all participating parents, and assent was obtained from all children at each timepoint. At each visit of the larger longitudinal study, children completed self-regulation tasks that were developmentally appropriate and mothers completed questionnaires. Mother–child dyads also completed interaction tasks. We used only the etch-a-sketch task described below and maternal report of demographic information for the current study. This task was administered in the second half of a 2-hour lab session and was videorecorded. The institutional review board at Virginia Tech approved procedures for the recoding of the videos for the current study. At each timepoint, mothers were paid $50 and children were given a small gift.

### Measure

#### Etch-A-Sketch Task

Mother–child dyads were seated at a child-size table in the lab for the etch-a-sketch task (Atzaba-Poria et al., 2014). Each dyad was given an etch-a-sketch and instructed to work together to draw assigned shapes with two knobs. Throughout the task, mothers were asked to only control one knob and children the other. At the 3-year-old timepoint, dyads drew a square followed by a circle, and the task lasted an average of 2.37 minutes (*SD* = 43; range = 2.00–4.87 minutes). At the 4-year-old timepoint, dyads drew a square followed by a house and finally a circle. The task lasted an average of 3.50 minutes (*SD* = 69; range = 2.05–6.68 minutes).

Two teams of coders who were unaware of research aims and hypotheses were independently trained and coded the video recordings in 30-second intervals. One team coded maternal emotion coaching and another team coded child emotion regulation. We used 30-second intervals for coding as they allowed meaningful measurement of maternal emotion coaching and child regulation within the flow of mother–child interactions.

#### Behavioral Coding of Maternal Emotion Coaching

We adapted an existing coding scheme to code maternal emotion coaching in the etch-a-sketch task (Dunsmore et al., 2013; Zhu et al., 2024). Emotion coaching is characterized by parents’ acceptance and validation of children’s emotions and assistance with teaching or problem-solving if needed (Gottman et al., 1996). Although behaviors such as labeling the child’s emotions or expressing empathy may be indicators of emotion coaching, emotion coaching as a construct is not a point or momentary behavior and the dynamic pacing of potential behavioral indicators within the interaction must be considered (Kehoe et al., 2020). Maternal emotion coaching was coded based on verbal and nonverbal behavioral indicators during each 30-second interval on a 4-point scale from 0 to 3: 0 = no coaching, 1 = acknowledges the child’s behavior or perspective in relation to the task, which demonstrates openness to the child’s experience (example: While the child and parent turn their knobs, parent says “Tell me when you want me to stop.”), 2 = shows acceptance of the child’s emotion (example: Child taps the screen and makes a growling sound and parent says “Take a couple deep breaths.”), and 3 = demonstrates full-blown emotion coaching, such as showing empathy and labeling the child’s emotions. When there was evidence for more than one point on the scale (e.g., an indicator of a score of 1 early in the interval and an indicator of a score of 3 later in the interval) then the higher score was coded. Coaching of positive and negative emotions was coded separately. Thus, there were codes for coaching positive emotions and for coaching negative emotions at each interval.

Coding teams held group consensus meetings at least biweekly to resolve discrepancies and maintain reliability. Each month, observer drift was checked. Reliability was assessed on 30.8% of codable intervals. According to Koo and Li (2016), moderate interrater reliability is demonstrated by ICCs of .50 to .75, good interrater reliability by ICCs ranging from .75 to .90, and excellent interrater reliability by ICCs greater than .90. Reliability was good for coaching of positive emotions (across timepoints, average ICC_weighted_ = .800). Reliability was moderate for coaching of negative emotions (across timepoints, average ICC_weighted_ = .561). Also, variability in codes influences how much disagreements between coders diminish ICCs (Lee et al., 2012). For coaching of negative emotions, only 1.59% of coded intervals had nonzero scores. Therefore, we deemed reliability to be adequate for coaching of negative emotions.

#### Behavioral Coding of Child Emotion Regulation

We adapted an existing coding scheme to code child emotion regulation in the etch-a-sketch at each timepoint (Calkins et al., 2004; Smith et al., 2004). Child emotion regulation was coded on three indices—noncompliance, engagement, and frustration—for each 30-second interval. Child noncompliance with mothers’ instructions was coded on a 5-point scale: n/a = no situations where child needed to comply, 1 = compliant, 2 = somewhat compliant, 3 = moderately noncompliant, and 4 = very noncompliant. Child engagement, meaning the amount of time the child spent on- or off-task, was coded on a 4-point scale: 1 = not engaged at all, 2 = slightly engaged, 3 = mostly engaged, and 4 = completely engaged. Child frustration was also coded on a 4-point scale: 1 = none, 2 = a little, 3 = some, and 4 = a lot. Group consensus meetings were held weekly or biweekly to resolve discrepancies. Reliability was assessed on 31.1% of codable videos and was good for all three indices (across timepoints, average ICC_weighted_ = .847, .825, and .843 for noncompliance, engagement, and frustration, respectively).

### Data Screening and Reduction

Upon completion of the two sets of coding, we conducted a two-step data screening. In Step 1, we double-checked documented starting times between the two coding teams for each included dyad at each timepoint. Dyads (*n* = 5 for age 3 timepoint and *n* = 33 for age 4 timepoint) were excluded from the sample if their video recordings had no on-screen timestamps, thus making it impossible to precisely match 30-second intervals across coding teams. The coding teams worked independently and used slightly different approaches for starting times, resulting in a few dyads (*n* = 4 for age 3 timepoint and *n* = 1 for age 4 timepoint) with mismatched intervals (discrepancies of more than 10 seconds). These dyads were also excluded from the samples. The final sample for the 3-year-old timepoint (*n* = 208) included 95.9% of the mother–child dyads who had completed the etch-a-sketch task at age 3, and the final sample for the 4-year-old timepoint (*n* = 227) included 87.0% of the mother–child dyads who had completed the etch-a-sketch task at age 4. In step 2, we examined the coded intervals across two sets of coding and included all intervals with both maternal emotion coaching codes and child regulation codes. The total number of intervals for dyads included in the sequential analysis ranged from two to eight for the age 3 timepoint and two to six for the age 4 timepoint.

Then, we examined code frequencies at each timepoint to inform data reduction. Supplemental Tables S1 and S2 show the frequency distributions. Sequential analysis requires the identification of given and target events. The goal of data reduction was to maximize the occurrence of these focal events, eliminate structural zeroes, and obtain more reliable statistical parameters for our subsequent sequential analyses (Fields-Olivieri & Cole, 2019; Van Bommel et al., 2019). Based on the frequency distributions, we collapsed maternal emotion coaching into absence (0) and presence (1, 2, 3) events. Similarly, we collapsed child noncompliance into compliance (1), presence of noncompliance (2, 3, 4), and n/a (no instructions for child to comply with) events. We collapsed child engagement into complete engagement (4) and presence of disengagement (1, 2, 3) events and child frustration into absence of frustration (1) and presence of frustration (2, 3, 4) events. Each set of events is mutually exclusive and exhaustive.

### Analytic Plan

We conducted lag-sequential analyses with lag 1 transitions using the software Generalized Sequential Querier (GSEQ Version 5.1.23; Bakeman & Quera, 2016) to test our two aims. Data were pooled across all dyads at each timepoint. To test how child emotion regulation contingently occurred following maternal emotion coaching (Aim 1), we specified the presence of maternal emotion coaching as the given event (lag 0) and the presence of child emotion regulation as the target event (lag 1). Maternal coaching of positive and negative emotions as well as children’s three emotion regulation indices (noncompliance, engagement, frustration) were tested separately, resulting in six sequential tests. To test how maternal emotion coaching contingently occurred following child emotion regulation (Aim 2), we specified the presence of child regulation (noncompliance, engagement, and frustration, one at a time) as the given event and presence of maternal coaching (coaching positive and coaching negative, one at a time) as the target event.

Three parameters were interpreted in the sequential analysis. First, joint frequency refers to the total count observed for each interactive sequence when the given event at one interval was followed by the target event at the next interval. When the count was less than the recommended cut-off of five (Bakeman & Quera, 2011), we treated the following remaining contingency indices as missing. Otherwise, we proceeded with reporting the two other parameters. Adjusted residuals indicate whether observed frequencies of sequences significantly deviated from expected frequencies. Odds ratios with 95% confidence intervals (CIs) measure effect size and indicate statistical significance. Odds ratios can vary from 0 to infinity. Statistically significant odds ratios above 1 indicate that the target event is more likely to occur in the interval after the given event (lag 1) whereas statistically significant odds ratios below 1 indicate that the target event is less likely to occur in the interval after the given event. Odds ratios between 1.25 and 2.00 for positive associations (0.50–0.80 for negative associations) indicate weak effect sizes; those between 2.00 and 3.00 (0.33–0.50) indicate moderate effect sizes; and those over 3.00 (less than 0.33) indicate strong effect sizes (Bakeman & Quera, 2011; Haddock, 1998).

## Results

### Preliminary Analyses

We conducted *t*-tests to examine child gender differences of studied variables at the dyad level across intervals and found that at the 3-year-old timepoint, girls were more compliant than boys (*t*(224) = 2.10, *p* = .037, Cohen’s *d* = 0.48). At the 4-year-old timepoint, mothers of girls displayed higher levels of coaching of negative emotions than mothers of boys (*t*(206) = 2.18, *p* = .031, Cohen’s *d* = 0.17). There were no other significant child gender differences (*p*s ≥ .110).

### Reciprocal Exchanges in Maternal Emotion Coaching and Child Emotion Regulation at Age 3 Years

Please see Table 2 for lag-sequential analysis results examining the presence of child emotion regulation following maternal emotion coaching and vice versa. In the presence of maternal coaching of positive emotions compared to its absence, children were significantly more likely to show compliance. The converse relation stood as well, such that maternal coaching of positive emotions was significantly more likely to be present following children’s compliance compared to their noncompliance. These findings are consistent with hypotheses. Significant associations in either direction were not found for maternal coaching of positive emotion with child engagement or frustration.

**Table 2:** Sequential analysis (Lag = 1) of reciprocal exchanges in maternal emotion coaching and child emotion regulation at age 3 years

Association	Given event	Target event	Given event frequency	Target event frequency	Joint frequency	Adjusted residual	Odds ratio	Effect size	95% CIs
MCoP ↕ Child emotion regulation	Presence of MCoP	Compliance	354	454	246	3.74	1.78**	Weak	[1.32, 2.42]
	Compliance	Presence of MCoP	445	311	209	2.79	1.55*	Weak	[1.14, 2.10]
	Presence of MCoP	Engagement	354	556	282	1.85	1.39		[0.98, 1.96]
	Engagement	Presence of MCoP	597	311	262	1.16	1.26		[0.85, 1.86]
	Presence of MCoP	Presence of frustration	354	103	47	− 0.70	0.86		[0.57, 1.31]
	Presence of frustration	Presence of MCoP	93	311	32	− 1.77	0.66		[0.42, 1.05]
MCoN ↕ Child emotion regulation	Presence of MCoN	Compliance	17	454	10	− 0.33	0.85		[0.32, 2.26]
	Compliance	Presence of MCoN	445	17	8	− 1.23	0.55		[0.21, 1.45]
	Presence of MCoN	Engagement	17	556	14	0.56	1.43		[0.41, 5.03]
	Engagement	Presence of MCoN	597	17	14	0.00	1.00		[0.28, 3.53]
	Presence of MCoN	Presence of frustration	17	103	6	2.52	3.44*	Strong	[1.24, 9.50]
	Presence of frustration	Presence of MCoN	93	17	5	2.07	2.94*	Moderate	[1.01, 8.53]

*MCoP* = maternal coaching of positive emotion, *MCoN* = maternal coaching of negative emotion. *p < .05, **p < .01

Joint frequency values were limited (range = 5–14) for reciprocal relations between child emotion regulation and maternal coaching of negative emotions. Nevertheless, following the presence of maternal coaching of negative emotions compared to its absence, children were more likely to display frustration. Conversely, when child frustration was present compared to absent, mothers were subsequently more likely to coach negative emotions. These findings contradicted hypotheses. Significant associations in either direction were not found for maternal coaching of negative emotion with child noncompliance or engagement.

### Reciprocal Exchanges in Maternal Emotion Coaching and Child Emotion Regulation at Age 4 Years

Please see Table 3 for lag-sequential analysis results examining the presence of child emotion regulation following maternal emotion coaching and vice versa. When mothers coached positive emotions compared to its absence, two of the three child regulation codes were subsequently significantly related: children were more likely to show compliance and engagement. No significant associations were detected for either maternal coaching of positive emotions and child frustration or the reverse directions. Maternal coaching of positive emotions was not significantly more or less likely following child compliance, engagement, or frustration.

**Table 3:** Sequential analysis (Lag = 1) of reciprocal exchanges in maternal emotion coaching and child emotion regulation at age 4 years

Association	Given event	Target event	Given event frequency	Target event frequency	Joint frequency	Adjusted residual	Odds ratio	Effect size	95% CIs
MCoP ↕ Child emotion regulation	Presence of MCoP	Compliance	421	543	263	2.26	1.36*	Weak	[1.04, 1.76]
	Compliance	Presence of MCoP	576	340	218	1.01	1.15		[0.87, 1.52]
	Presence of MCoP	Engagement	421	607	298	3.17	1.56**	Weak	[1.18, 2.06]
	Engagement	Presence of MCoP	649	340	244	0.96	1.15		[0.86, 1.55]
	Presence of MCoP	Presence of frustration	421	77	27	− 1.89	0.63		[0.39, 1.02]
	Presence of frustration	Presence of MCoP	62	340	20	− 0.73	0.81		[0.47, 1.41]
MCoN ↕ Child emotion regulation	Presence of MCoN	Compliance	11	543	8	0.97	1.91		[0.50, 7.24]
	Compliance	Presence of MCoN	576	13	10	1.12	2.06		[0.56, 7.54]
	Presence of MCoN	Engagement	11	607	7	− 0.12	0.93		[0.27, 3.19]
	Engagement	Presence of MCoN	649	13	10	0.56	1.44		[0.39, 5.29]
	Presence of MCoN	Presence of frustration	11	77	0	—	—	—	—
	Presence of frustration	Presence of MCoN	62	13	2	—	—	—	—

*MCoP* = maternal coaching of positive emotion, *MEoN* = maternal coaching of negative emotion. *p < .05

Similar to the 3-year-old timepoint, joint frequencies of child emotion regulation and maternal coaching of negative emotions were limited (range = 0–10). Only four associations could be tested (child compliance following maternal coaching of negative emotions, the reverse maternal coaching of negative emotions following child compliance, child engagement following maternal coaching of negative emotions, and the reverse maternal coaching of negative emotions following child engagement) and neither was significant.

## Discussion

Our purpose was to examine dynamic emotion socialization processes during early childhood by conducting sequential analyses to investigate temporal sequences between maternal emotion coaching and child emotion regulatory behaviors during mother–child interactions. Results demonstrated reciprocal sequences at age 3 and parent-driven sequences at age 4.

For age 3, we found two significant bidirectional sequences. First was the reciprocal sequential contingency between maternal coaching of positive emotions and children’s compliance. When mothers encouraged positive emotions, their child was subsequently more likely to show compliance. Additionally, when children complied, their mother was subsequently more likely to encourage their positive emotions. These transactions suggest a virtuous cycle of within-interaction positivity (Hummel et al., 2016). When mothers encourage their child’s perspective or positive emotions, the child may feel validated and therefore display more collaborative behaviors including compliance and responsiveness to the mother (Yi et al., 2016). These child-collaborative behaviors may in turn provide more opportunities for maternal emotion coaching behaviors.

The second bidirectional sequence at age 3 was the reciprocal sequential contingency between maternal coaching of negative emotions and child frustration. When mothers encouraged negative emotions, their child was subsequently more likely to display frustration. When children showed frustration, their mother was subsequently more likely to encourage negative emotions. The etch-a-sketch task can elicit emotions, particularly with more challenging shapes. In challenging circumstances, mothers’ validation of children’s negativity may communicate that emotions are acceptable and foster an emotionally supportive context where the child may experience frustration without being overwhelmed and may practice socially appropriate emotion management with mothers’ guidance (Dunsmore et al., 2013; Katz et al., 2012). Children’s more open displays of frustration may then further lead to mothers’ encouragement as the child responds with an invited expression that the mother considers appropriate.

For age 4, we found significant parent-driven within-interaction sequences for two child emotion regulation indices—compliance and engagement. After mothers encouraged children’s positive emotions, children were subsequently more likely to show compliance and engagement. Findings support the role of maternal coaching of positive emotions in fostering children’s emotion regulation (Ramakrishnan et al., 2019). In contrast to age 3, we did not find significant child-driven sequences at age 4. Also, we were not able to test all sequences for maternal coaching of negative emotions due to its rare occurrence. Perhaps 4-year-old children’s developmental advancement in self-regulation allows mothers to take a more passive role and leave more space for their child’s autonomy in regulating their task behavior and emotions (Feng et al., 2017). Supporting this interpretation, post hoc paired *t*-tests using composited scores for each child regulatory behavior across intervals at each timepoint showed a significant increase in child engagement from age 3 to age 4 (*t*(180) = 2.84, *p* = .005, Cohen’s *d* = .43), though child compliance and frustration did not show significant differences across timepoints (*p*s > .095). Additionally, despite adding a challenging picture, the difficulty level of the etch-a-sketch task at age 4 may be within reach for more dyads than was the case at age 3. Development of fine motor skills and cognitive and socio-emotional development during early childhood may contribute to children’s ability to maintain their engagement with the task without mothers’ co-regulatory assistance (Brown, 2010; Saarni et al., 2006).

The significant findings for maternal coaching of positive emotions at both timepoints suggest that tasks like the etch-a-sketch task in our study may be promising for emotion socialization research by placing parent–child dyads in situations that have parallels to daily lives and that elicit unprompted socialization of positive emotions (Zhu et al., 2024). This is important because coaching of positive and negative emotions may serve different functions. Parental coaching of negative emotions such as validating their child’s frustration or problem-solving the anger-eliciting circumstance may directly increase the child’s emotional awareness and teach appropriate skills to regulate negative emotions (Eisenberg et al., 1998). Coaching of positive emotions such as mirroring a child’s excitement cultivates the child’s positive emotional experiences, which may indirectly foster regulation of negative emotions and enhance children’s psychological adjustment (Doan et al., 2023; Fredrickson, 1998).

Our study has key strengths. First, including two timepoints when children were aged 3 and 4 showcased child behavioral and emotional development during early childhood. Second, behavioral observation of mother–child interactions on the etch-a-sketch task functioned as an ecologically valid measure of mothers’ spontaneous emotion coaching. Coding of maternal emotion coaching and child emotion regulation with two independent teams reduced the risk of coding bias. Coding of both positive and negative emotion coaching broadened knowledge of emotion coaching processes. Third, the sequential analyses examined within-interaction relations, contributing to the understanding of emotion socialization as a process that unfolds during mother–child interactions (Bakeman & Quera, 2011; Eisenberg et al., 1998; Wang, 2023). Our exploration of both parent-driven and child-driven sequences contributed to studying children’s agency in their socialization (Sperling & Repetti, 2018).

We recognize some limitations that inspire future directions. First, though we had a relatively large sample size, our samples were limited to mother–child dyads from the southeastern United States who mostly identified as White and had completed college education or higher. Future research with culturally, ethnoracially, and socioeconomically diverse samples is important to determine generalizability. To comprehensively understand emotion socialization processes within the family setting, future studies need to include fathers and other family members (Eisenberg, 2020). Second, future studies including multiple tasks will provide a more comprehensive picture of emotion socialization processes. Our use of sequential analyses required categorizing study variables during data reduction. This categorization limited the variable range and provided insufficient power to test findings within demographic groups. In addition, we examined next-interval contingencies between maternal emotion coaching and child regulation. Future research using other analytic strategies such as grid-sequence analysis and multilevel survival analysis (e.g., Lougheed et al., 2019, 2020) may provide a more nuanced understanding of emotion coaching dynamics over a longer course of interactions in real time incorporating the timing of events.

In conclusion, we found bidirectional sequences of maternal emotion coaching of positive and negative emotions with child emotion regulation for 3-year-olds, and parent-driven sequences of maternal coaching of positive emotions followed by child emotion regulatory compliance and engagement for 4-year-olds. Findings suggest the intricacy of dynamic emotion socialization transactions in relation to child emotion regulation.

## Supplementary Information

Below is the link to the electronic supplementary material.

Supplementary file1 (DOCX 46.0 KB)

## Additional Information

### Availability of data and material

The data that support the findings of this study are available in the Open Science Framework repository, https://osf.io/q65dy/?view_only=68311a80a9114ce7965b3445ae3a651d.
