# Supplementary material for: Maternal Emotion Coaching and Child Emotion Regulation: Within-Interaction Sequences in Early Childhood
Source: Affect Sci. 2025 Jan 11;6(2):214–23. doi: 10.1007/s42761-024-00285-7 (PMC12209066; doi:10.1007/s42761-024-00285-7)
Supplement: Supplementary file 1 — Supplementary file1 (DOCX 46.0 KB) [file 42761_2024_285_MOESM1_ESM.docx]

**Maternal Emotion Coaching and Child Emotion Regulation: Within-Interaction Sequences in Early Childhood**

**Table S1**. Age 3 Timepoint Frequencies (Percentages) for Study Variables at 30-second Intervals

| **Variable** | **Scale** | **Code** | **1^st^ Interval** | **2^nd^ Interval** | **3^rd^ Interval** | **4^th^ Interval** | **5^th^ Interval** | **6^th^ Interval** | **7^th^ Interval** | **8^th^ Interval** |
| --- | --- | --- | --- | --- | --- | --- | --- | --- | --- | --- |
|  |  |  | (n=208; 100%) | (n=208; 100%) | (n=203; 97.60%) | (n=193; 92.79%) | (n=83; 39.90%) | (n=30; 9.74%) | (n=7; 3.37%) | (n=1; 0.48%) |
| Maternal ENC of Positive Emotions | initial 4-point scale | 0 (no ENC) | 90 (43.27) | 71 (34.13) | 146 (70.19) | 120 (57.69) | 48 (23.08) | 22 (10.58) | 4 (1.92) | 0 (0.00) |
|  |  | 1 (ENC of perspective) | 114 (54.81) | 125 (60.10) | 49 (23.56) | 67 (32.21) | 29 (13.94) | 8 (3.85) | 3 (1.44) | 1 (0.48) |
|  |  | 2 (ENC of emotion) | 4 (1.92) | 11 (5.29) | 5 (2.40) | 6 (2.88) | 6 (2.88) | 0 (0.00) | 0 (0.00) | 0 (0.00) |
|  |  | 3 (coaching) | 0 (0.00) | 1 (0.48) | 0 (0.00) | 0 (0.00) | 0 (0.00) | 0 (0.00) | 0 (0.00) | 0 (0.00) |
|  | post-pooling | absence (0) | 90 (43.27) | 71 (34.13) | 146 (70.19) | 120 (57.69) | 48 (23.08) | 22 (10.58) | 4 (1.92) | 0 (0.00) |
|  |  | presence (1/2/3) | 118 (56.73) | 137 (65.87) | 54 (24.96) | 73 (35.09) | 35 (16.82) | 8 (3.85) | 3 (1.44) | 1 (0.48) |
| Maternal ENC of Negative Emotions | initial 4-point scale | 0 (no ENC) | 203 (97.60) | 203 (97.60) | 197 (94.71) | 188 (90.38) | 80 (38.46) | 29 (13.94) | 6 (2.88) | 1 (0.48) |
|  |  | 1 (ENC of perspective) | 0 (0.00) | 0 (0.00) | 0 (0.00) | 0 (0.00) | 0 (0.00) | 0 (0.00) | 0 (0.00) | 0 (0.00) |
|  |  | 2 (ENC of emotion) | 5 (2.40) | 4 (1.92) | 2 (0.96) | 5 (2.40) | 1 (0.48) | 1 (0.48) | 1 (0.48) | 0 (0.00) |
|  |  | 3 (coaching) | 0 (0.00) | 1 (0.48) | 1 (0.48) | 0 (0.00) | 2 (0.96) | 0 (0.00) | 0 (0.00) | 0 (0.00) |
|  | post-pooling | absence (0) | 203 (97.60) | 203 (97.60) | 197 (94.71) | 188 (90.38) | 80 (38.46) | 29 (13.94) | 6 (2.88) | 1 (0.48) |
|  |  | presence (1/2/3) | 5 (2.40) | 5 (2.40) | 3 (1.44) | 5 (2.40) | 3 (1.44) | 1 (0.48) | 1 (0.48) | 0 (0.00) |
| Child Emotion Regulation – Noncompliance | initial 5-point scale | n/a (not applicable) | 17 (8.17) | 19 (9.13) | 27 (12.98) | 21 (10.10) | 14 (6.73) | 3 (1.44) | 1 (0.48) | 0 (0.00) |
|  |  | 1 (compliant) | 121 (58.17) | 125 (60.10) | 123 (59.13) | 133 (63.94) | 51 (24.52) | 19 (9.13) | 3 (1.44) | 0 (0.00) |
|  |  | 2 (somewhat compliant) | 62 (29.81) | 54 (25.96) | 49 (23.56) | 35 (16.83) | 13 (6.25) | 3 (1.44) | 0 (0.00) | 1 (0.48) |
|  |  | 3 (moderately noncompliant) | 6 (2.88) | 8 (3.85) | 4 (1.92) | 4 (1.92) | 5 (2.40) | 3 (1.44) | 2 (0.96) | 0 (0.00) |
|  |  | 4 (very noncompliant) | 2 (0.96) | 2 (0.96) | 0 (0.00) | 0 (0.00) | 0 (0.00) | 2 (0.96) | 1 (0.48) | 0 (0.00) |
|  | post-pooling | n/a | 17 (8.17) | 19 (9.13) | 27 (12.98) | 21 (10.10) | 14 (6.73) | 3 (1.44) | 1 (0.48) | 0 (0.00) |
|  |  | compliance (1) | 121 (58.17) | 125 (60.10) | 123 (59.13) | 133 (63.94) | 51 (24.52) | 19 (9.13) | 3 (1.44) | 0 (0.00) |
|  |  | presence of noncompliance (2/3/4) | 70 (33.65) | 64 (30.77) | 53 (25.48) | 39 (18.75) | 18 (8.65) | 8 (3.84) | 3 (1.44) | 1 (0.48) |
| Child Emotion Regulation – Engagement | initial 4-point scale | 1 (not engaged at all) | 0 (0.00) | 0 (0.00) | 0 (0.00) | 2 (0.96) | 0 (0.00) | 3 (1.44) | 1 (0.48) | 0 (0.00) |
|  |  | 2 (slightly engaged) | 2 (0.96) | 3 (1.44) | 8 (3.85) | 9 (4.33) | 4 (1.92) | 1 (0.48) | 0 (0.00) | 0 (0.00) |
|  |  | 3 (mostly engaged) | 24 (11.54) | 31 (14.90) | 41 (19.71) | 45 (21.63) | 16 (7.69) | 4 (1.92) | 1 (0.48) | 0 (0.00) |
|  |  | 4 (completely engaged) | 182 (87.50) | 174 (83.65) | 154 (74.04) | 137 (65.87) | 63 (30.29) | 22 (10.58) | 5 (2.40) | 1 (0.48) |
|  | post-pooling | engagement (4) | 182 (87.50) | 174 (83.65) | 154 (74.04) | 137 (65.87) | 63 (30.29) | 22 (10.58) | 5 (2.40) | 1 (0.48) |
|  |  | presence of disengagement (1/2/3) | 26 (12.50) | 34 (16.34) | 49 (23.56) | 56 (26.92) | 20 (9.61) | 8 (3.84) | 2 (0.96) | 0 (0.00) |
| Child Emotion Regulation – Frustration | initial 4-point scale | 1 (none) | 192 (92.31) | 181 (87.02) | 172 (82.69) | 168 (80.77) | 71 (34.13) | 24 (11.54) | 5 (2.40) | 1 (0.48) |
|  |  | 2 (a little) | 12 (5.77) | 24 (11.54) | 30 (14.42) | 23 (11.06) | 10 (4.81) | 5 (2.40) | 2 (0.96) | 0 (0.00) |
|  |  | 3 (some) | 3 (1.44) | 2 (0.96) | 1 (0.48) | 2 (0.96) | 2 (0.96) | 1 (0.48) | 0 (0.00) | 0 (0.00) |
|  |  | 4 (a lot) | 1 (0.48) | 1 (0.48) | 0 (0.00) | 0 (0.00) | 0 (0.00) | 0 (0) | 0 (0.00) | 0 (0.00) |
|  | post-pooling | no frustration (1) | 192 (92.31) | 181 (87.02) | 172 (82.69) | 168 (80.77) | 71 (34.13) | 24 (11.54) | 5 (2.40) | 1 (0.48) |
|  |  | presence of frustration (2/3/4) | 16 (7.69) | 27 (12.98) | 31 (14.90) | 25 (12.02) | 12 (5.77) | 6 (2.88) | 2 (0.96) | 0 (0.00) |

Notes. ENC: encouragement.

**Table S2**. Age 4 Timepoint Frequencies (Percentages) for Study Variables at 30-second Intervals

| **Variable** | **Scale** | **Code** | **1^st^ Interval** | **2^nd^ Interval** | **3^rd^ Interval** | **4^th^ Interval** | **5^th^ Interval** | **6^th^ Interval** |
| --- | --- | --- | --- | --- | --- | --- | --- | --- |
|  |  |  | (n=227; 100%) | (n=227; 100%) | (n=226; 99.56%) | (n=205; 90.31%) | (n=168; 74.01%) | (n=103; 45.37%) |
| Maternal ENC of Positive Emotions | initial 4-point scale | 0 (no ENC) | 81 (35.68) | 99 (43.61) | 159 (70.04) | 143 (63.00) | 107 (47.14) | 78 (34.36) |
|  |  | 1 (ENC of perspective) | 137 (60.35) | 111 (48.90) | 54 (23.79) | 55 (24.23) | 58 (25.55) | 20 (8.81) |
|  |  | 2 (ENC of emotion) | 9 (3.96) | 17 (7.49) | 11 (4.85) | 7 (3.08) | 3 (1.32) | 5 (2.20) |
|  |  | 3 (coaching) | 0 (0.00) | 0 (0.00) | 0 (0.00) | 0 (0.00) | 0 (0.00) | 0 (0.00) |
|  | post-pooling | absence (0) | 81 (35.68) | 99 (43.61) | 159 (70.04) | 143 (63.00) | 107 (47.14) | 78 (34.36) |
|  |  | presence (1/2/3) | 146 (64.31) | 128 (56.39) | 65 (28.64) | 62 (27.31) | 61 (26.87) | 25 (11.01) |
| Maternal ENC of Negative Emotions | initial 4-point scale | 0 (no ENC) | 226 (99.56) | 227 (100.00) | 219 (96.48) | 202 (88.99) | 164 (72.25) | 102 (44.93) |
|  |  | 1 (ENC of perspective) | 0 (0.00) | 0 (0.00) | 0 (0.00) | 0 (0.00) | 0 (0.00) | 0 (0.00) |
|  |  | 2 (ENC of emotion) | 1 (0.44) | 0 (0.00) | 4 (1.76) | 3 (1.32) | 4 (1.76) | 0 (0.00) |
|  |  | 3 (coaching) | 0 (0.00) | 0 (0.00) | 1 (0.44) | 0 (0.00) | 0 (0.00) | 1 (0.44) |
|  | post-pooling | absence (0) | 226 (99.56) | 227 (100.00) | 219 (96.48) | 202 (88.99) | 164 (72.25) | 102 (44.93) |
|  |  | presence (1/2/3) | 1 (0.44) | 0 (0.00) | 5 (2.20) | 3 (1.32) | 4 (1.76) | 1 (0.44) |
| Child Emotion Regulation – Noncompliance | initial 5-point scale | n/a (not applicable) | 5 (2.20) | 11 (4.85) | 2 (0.88) | 8 (3.52) | 5 (2.20) | 8 (3.52) |
|  |  | 1 (compliant) | 145 (63.88) | 140 (61.67) | 137 (60.35) | 118 (51.98) | 97 (42.73) | 51 (22.47) |
|  |  | 2 (somewhat compliant) | 50 (22.03) | 48 (21.15) | 53 (23.35) | 50 (22.03) | 45 (19.82) | 31 (13.66) |
|  |  | 3 (moderately noncompliant) | 27 (11.89) | 27 (11.89) | 33 (14.54) | 29 (12.78) | 21 (9.25) | 12 (5.29) |
|  |  | 4 (very noncompliant) | 0 (0.00) | 1 (0.44) | 1 (0.44) | 0 (0.00) | 0 (0.00) | 1 (0.44) |
|  | post-pooling | n/a | 5 (2.20) | 11 (4.85) | 2 (0.88) | 8 (3.52) | 5 (2.20) | 8 (3.52) |
|  |  | compliance (1) | 145 (63.88) | 140 (61.67) | 137 (60.35) | 118 (51.98) | 97 (42.73) | 51 (22.47) |
|  |  | presence of noncompliance (2/3/4) | 77 (33.92) | 76 (33.48) | 87 (38.33) | 79 (34.81) | 66 (29.07) | 44 (19.39) |
| Child Emotion Regulation – Engagement | initial 4-point scale | 1 (not engaged at all) | 0 (0.00) | 0 (0.00) | 0 (0.00) | 1 (0.44) | 0 (0.00) | 2 (0.88) |
|  |  | 2 (slightly engaged) | 1 (0.44) | 2 (0.88) | 4 (1.76) | 4 (1.76) | 7 (3.08) | 3 (1.32) |
|  |  | 3 (mostly engaged) | 57 (25.11) | 47 (20.70) | 71 (31.28) | 73 (32.16) | 66 (29.07) | 42 (18.50) |
|  |  | 4 (completely engaged) | 169 (74.45) | 178 (78.41) | 151 (66.52) | 127 (55.95) | 95 (41.85) | 56 (24.67) |
|  | post-pooling | engagement (4) | 169 (74.45) | 178 (78.41) | 151 (66.52) | 127 (55.95) | 95 (41.85) | 56 (24.67) |
|  |  | presence of disengagement (1/2/3) | 58 (25.55) | 49 (21.58) | 75 (33.04) | 78 (34.36) | 73 (32.15) | 47 (20.70) |
| Child Emotion Regulation – Frustration | initial 4-point scale | 1 (none) | 213 (93.83) | 215 (94.71) | 208 (91.63) | 187 (82.38) | 154 (67.84) | 88 (38.77) |
|  |  | 2 (a little) | 11 (4.85) | 11 (4.85) | 17 (7.49) | 14 (6.17) | 10 (4.41) | 12 (5.29) |
|  |  | 3 (some) | 3 (1.32) | 0 (0.00) | 1 (0.44) | 4 (1.76) | 4 (1.76) | 3 (1.32) |
|  |  | 4 (a lot) | 0 (0.00) | 1 (0.44) | 0 (0.00) | 0 (0.00) | 0 (0.00) | 0 (0.00) |
|  | post-pooling | no frustration (1) | 213 (93.83) | 215 (94.71) | 208 (91.63) | 187 (82.38) | 154 (67.84) | 88 (38.77) |
|  |  | presence of frustration (2/3/4) | 14 (6.17) | 12 (5.29) | 18 (7.93) | 18 (7.93) | 14 (6.17) | 15 (6.61) |
